# GCLink: a graph contrastive link prediction framework for gene regulatory network inference

**DOI:** 10.1093/bioinformatics/btaf074

**Published:** 2025-02-17

**Authors:** Weiming Yu, Zerun Lin, Miaofang Lan, Le Ou-Yang

**Affiliations:** Guangdong Provincial Key Laboratory of Intelligent Information Processing and Shenzhen Key Laboratory of Media Security, College of Electronics and Information Engineering, Shenzhen University, Shenzhen 518060, China; Guangdong Provincial Key Laboratory of Intelligent Information Processing and Shenzhen Key Laboratory of Media Security, College of Electronics and Information Engineering, Shenzhen University, Shenzhen 518060, China; Guangdong Provincial Key Laboratory of Intelligent Information Processing and Shenzhen Key Laboratory of Media Security, College of Electronics and Information Engineering, Shenzhen University, Shenzhen 518060, China; Guangdong Laboratory of Machine Perception and Intelligent Computing, Faculty of Engineering, Shenzhen MSU-BIT University, Shenzhen 518116, China

## Abstract

**Motivation:**

Gene regulatory networks (GRNs) unveil the intricate interactions among genes, pivotal in elucidating the complex biological processes within cells. The advent of single-cell RNA-sequencing (scRNA-seq) enables the inference of GRNs at single-cell resolution. However, the majority of current supervised network inference methods typically concentrate on predicting pairwise gene regulatory interaction, thus failing to fully exploit correlations among all genes and exhibiting limited generalization performance.

**Results:**

To address these issues, we propose a graph contrastive link prediction (GCLink) model to infer potential gene regulatory interactions from scRNA-seq data. Based on known gene regulatory interactions and scRNA-seq data, GCLink introduces a graph contrastive learning strategy to aggregate the feature and neighborhood information of genes to learn their representations. This approach reduces the dependence of our model on sample size and enhance its ability in predicting potential gene regulatory interactions. Extensive experiments on real scRNA-seq datasets demonstrate that GCLink outperforms other state-of-the-art methods in most cases. Furthermore, by pretraining GCLink on a source cell line with abundant known regulatory interactions and fine-tuning it on a target cell line with limited amount of known interactions, our GCLink model exhibits good performance in GRN inference, demonstrating its effectiveness in inferring GRNs from datasets with limited known interactions.

**Availability and implementation:**

The source code and data are available at https://github.com/Yoyiming/GCLink.

## 1 Introduction

Gene regulatory networks (GRNs) consist of the intricate regulatory interactions between transcription factors (TFs) and target genes, controlling the transcription processes and thus governing the cellular behaviors (Marbach *et al.* 2012). The reconstruction of GRNs plays a pivotal role in analyzing the complex mechanisms of gene expression with cells (Zhao *et al.* 2021). Recently, the rapid development of single-cell RNA-sequencing (scRNA-seq) has greatly enhanced the accumulation of gene expression data at the single-cell resolution (Kolodziejczyk *et al.* 2015). Unlike bulk transcriptomic data, scRNA-seq data can reflect the heterogeneity in expression among cells, rather than simply representing cellular expression level by averaging expression values over all the cells (Pratapa *et al.* 2020). Therefore, scRNA-seq data has the potential to reveal the intricate interaction between genes in an unprecedented resolution (Hwang *et al.* 2018). However, the fine-grained nature of scRNA-seq data also introduces numerous challenges. For example, the scRNA-seq data exhibits a high variability across cells (Wang *et al.* 2023) and demonstrates substantial sparsity caused by dropouts (Kharchenko *et al.* 2014), which poses significant difficulties for inferring GRNs from scRNA-seq data.

Despite facing these challenges, numerous computational methods have been proposed in the past years for inferring GRNs from scRNA-seq data. Pioneering methods construct co-expression networks for inferring GRNs based on correlation coefficients (Specht and Li 2017) or mutual information (Chan *et al.* 2017). However, gene interactions are inherently complex and nonlinear, making the use of correlation coefficients or mutual information alone insufficient to accurately capture these interactions. With the rapid development of machine learning algorithms, a number of machine learning-based methods have emerged for inferring GRNs. For example, PIDC (Chan *et al.* 2017) uses partial information decomposition to infer GRNs. Based on conditional mutual information, CN (Aghdam *et al.* 2015), and OIPCQ (Mahmoodi *et al.* 2021) can effectively enhance the accuracy of network inference and achieve optimal network selection by choosing specific thresholds. GENIE3 (Huynh-Thu *et al.* 2010) and GRNBoost2 (Moerman *et al.* 2019) are both tree-based machine learning methods for the inference of GRNs. By using Bayesian mixture model, wpLogicNet (Malekpour *et al.* 2023) can infer the directed GRN structures and significantly reduce the time complexity. Based on beta-variational autoencoder and structural equation model, Shu *et al.* proposed a deep learning-based method named DeepSEM to infer GRNs (Shu *et al.* 2021). DeepGRNCS (Lei *et al.* 2024) utilizes pre-trained multilayer perceptron (MLP) models to jointly infer GRNs across multiple cell subpopulations. Despite the significant results achieved by these methods, they continue to suffer from the major challenge of high false positive rates inherent in unsupervised learning methods (Yuan and Bar-Joseph 2019).

Recently, several deep learning-based supervised learning methods have been proposed to alleviate this problem. For example, CNNC (Yuan and Bar-Joseph 2019) converts gene expression matrix into images and utilizing convolutional neural network (CNN) to capture the regulatory interactions among genes. Building upon CNNC, DeepDRIM (Chen *et al.* 2021) takes into account the influence of neighboring genes on regulatory relationships, which more closely reflects the reality of biological processes. DeepMCL (Lin and Ou-Yang 2023) integrates multi-view scRNA-seq data and uses an attention mechanism to integrate the information provided by multiple neighbor genes. DeepFGRN (Gao *et al.* 2024) introduces a Generative Adversarial Network (GAN) and efficiently captures the relationships between regulators and target genes by combining it with CNNs. Based on MLPs, Kc *et al.* (2019) proposed a method named GNE to infer GRNs. Xu *et al.* (2023) enhanced GRN inference with gene expression motifs using a transformer-based method named STGRNS. Although these methods have achieved some success in GRN inference, they mainly rely on pairwise correlations between genes to predict potential regulatory relationships, overlooking the global information inherent in known regulatory interactions. As a result, they are dependent on the scale of the training data, sensitive to data noise, and have limited generalization ability.

In recent years, graph neural networks (GNNs) (Kipf and Welling 2017) have gained significant prominence in the analysis of graph-structure data. Various GNN-based methods have been proposed to address graph-based tasks, including node classification, graph classification, and link prediction (Zhang and Chen 2018). Chen and Liu framed the inference of GRNs as a link prediction problem and introduced GENELink (Chen and Liu 2022), a GNN-based method utilizing Graph Attention Network (GAT) (Velickovic *et al.* 2017) to infer GRNs. GAT can leverage the information aggregated from neighboring genes based on known regulatory interactions, and the self-attention mechanism enables GAT to adaptively adjust the influence of neighboring genes on the central gene. In light of the high dropout rates in scRNA-seq data, GNNLink (Mao *et al.* 2023) imputes the gene expression matrix before extracting gene features and utilizes Graph Convolutional Networks (GCNs) (Kipf and Welling 2017) for feature extraction. However, most GNN-based methods rely heavily on the abundance of known regulatory interactions. This reliance presents a challenge when handling scenarios where known regulatory interactions are limited or where the observed network contains noise.

To tackle the aforementioned challenges, we propose a graph contrastive link prediction model (GCLink) for inferring GRNs from scRNA-seq data. In particular, based on GNNs, we frame the inference of GRNs as a link prediction task, enabling information propagation through the capture of both local and global information to uncover complex regulatory relationships. Firstly, we use GAT to learn low-dimensional representations of genes. With the multi-head self-attention mechanism, GAT can assign importance weights to neighboring genes, thereby modulating the influence of these neighboring genes. Then, based on observed gene regulatory interactions, we generate another view of graph through graph augmentation, and introduce a contrastive loss to maximize the agreement of gene embeddings between these two graph views, which can acquire more precise low-dimensional embeddings of genes. Additionally, we adopt a pre-training strategy to make our model applicable to few-shot situations where limited known regulatory interactions are observed (You *et al.* 2020). We evaluate the performance of GCLink in inferring GRNs on various real scRNA-seq datasets. By comparing to six state-of-the-art methods, GCLink demonstrated superior performance in terms of AUROC and AUPRC scores. Furthermore, we conducted few-shot studies to validate the performance of GCLink when limited known regulatory interactions are available. Besides, we apply GCLink on the human embryonic stem cells dataset to infer cell-type specific GRNs, demonstrating its potential to uncover novel gene regulatory interactions.

## 2 Materials and methods

### 2.1 Problem formulation

A GRN can be represented as a graph G=(V,E), where V represents the node set and E denotes the edge set. The scRNA-seq data can be denoted as a gene expression matrix $X\in R^{M\times N}$, where *M* refers to the number of genes and *N* refers to the number of cells. Let $A\in R^{M\times M}$ denote the known gene regulatory interactions in the GRN, where $A_{ij}=1$ if $(v_{i},v_{j})\in E$ and $A_{ij}=0$ otherwise. Note that we consider TFs and target genes as nodes in G, while the observed edges in E represent the known regulatory interactions between TFs and genes. Our primary objective is to infer potential regulatory interactions between TFs and genes based on observed regulatory interactions, which can be formulated as a link prediction problem.

### 2.2 Graph augmentation

Given the sparsity nature of the observed GRN, to enhance the model’s ability to handle situations with limited known regulatory interactions, we utilize the strategies of retaining the original graph and randomly removing edges for graph augmentation. Retaining the original graph allows for maximum utilization of known information, while randomly removing edges enforces the model to adapt to extremely sparse scenario. To realize the graph augmentation, we leverage the *Identity* class and *EdgeRemoving* class provided by the PyGCL (Zhu *et al.* 2021) library. We denote G=(V,E) as the preserved graph which is identical to the original graph, and $\hat{G}=(V,\hat{E})$ as the perturbed graph which is randomly removed edges, where $\hat{E}$ refers to the set of remaining edges. In the subsequent sections, all symbols related to the perturbed graph will be denoted using a similar representation way.

### 2.3 Gene representation learning

We introduce a GNN-based encoder to extract the low-dimensional representation of genes from gene expression data. Specifically, we utilizes two GAT layers to learn gene representations, leveraging both the gene expression matrix and the adjacency matrix as inputs. Notably, the gene expression matrix is identical in both graph views, as we solely augment the topological structure of the graph. So the initial feature matrix of two views is the gene expression matrix *X* and the initial gene features are denoted as $X=\left\{g_{1},g_{2},\ldots ,g_{M}\right\},\;g_{i}\in R^{N}$. The adjacency matrix of the preserved graph is denoted as *A*, while the adjacency matrix of the perturbed graph is denoted as $\hat{A}$.

When learning the representations of genes, we first apply the self-attention mechanism α to genes and calculate the similarity coefficients between each gene and its neighboring genes:

(1) $$sim_{ij}=\alpha \left(\left[Wg_{i}||Wg_{j}\right]\right),j\in N_{i},$$

 

(2) $$\hat{sim_{ij}}=\alpha \left(\left[Wg_{i}||Wg_{j}\right]\right),j\in \hat{N_{i}},$$

where W is a learnable weight matrix shared for each gene; [·||·] refers to concatenation operation; $N_{i}=\{j\;|\;(v_{i},v_{j})\in E\}$ refers to the neighboring genes of gene *i* in the preserved graph and $\hat{N_{i}}=\{j\;|\;(v_{i},v_{j})\in \hat{E}\}$ refers to the neighboring genes of gene *i* in the perturbed graph. After that, we normalize them using softmax function to obtain the final attention scores:

(3) $$att_{ij}=\frac{\;\text{exp}(\text{LeakyReLu}(sim_{ij}))}{\sum_{n\in N_{i}}\;\text{exp}\;(\text{LeakyReLu}(sim_{in}))},$$

 

(4) $$\hat{att_{ij}}=\frac{\;\text{exp}(\text{LeakyReLu}(\hat{sim_{ij}}))}{\sum_{n\in \hat{N_{i}}}\;\text{exp}\;(\text{LeakyReLu}(\hat{sim_{in}}))},$$

We utilize a multi-head attention mechanism to improve the stability of the learning process of self-attention and perform a weighted summation of gene information:

(5) $$h_{i}^{\left(1\right)}={\left|\right|}_{k=1}^{K}\sigma \left(\sum_{j\in N_{i}}att_{ij}^{(1)k}W_{1}^{k}g_{j}\right),$$

 

(6) $${\hat{h}}_{i}^{\left(1\right)}={\left|\right|}_{k=1}^{K}\sigma \left(\sum_{j\in \hat{N_{i}}}{\hat{\mathrm{att}}}_{ij}^{(1)k}W_{1}^{k}g_{j}\right),$$

where || is the concatenation operation, σ(·) is the activation function, $att_{ij}^{(1)k}$ and ${\hat{\mathrm{att}}}_{ij}^{(1)k}$ refer to the *k*th normalized attention scores of the preserved graph and the perturbed graph respectively, and $W_{1}^{k}$ is the corresponding weight matrix. $h_{i}^{\left(1\right)}$ and ${\hat{h}}_{i}^{\left(1\right)}$ are the output embeddings of the first GAT layer. We then fed them into the second GAT layer to aggregate information again:

(7) $$h_{i}^{\left(2\right)}=\sigma \left(\frac{1}{K}\sum_{k=1}^{K}\sum_{j\in N_{i}}att_{ij}^{(2)k}W_{2}^{k}h_{j}^{\left(1\right)}\right),$$

 

(8) $${\hat{h}}_{i}^{\left(2\right)}=\sigma \left(\frac{1}{K}\sum_{k=1}^{K}\sum_{j\in \hat{N_{i}}}{\hat{\mathrm{att}}}_{ij}^{(2)k}W_{2}^{k}{\hat{h}}_{j}^{\left(1\right)}\right).$$

Due to the second GAT layer is the last layer of GAT, we use averaging instead of concatenating. So far, we have obtained the representation for gene *i* in the two different graphs, namely $h_{i}^{\left(2\right)}$ and ${\hat{h}}_{i}^{\left(2\right)}$.

### 2.4 Graph contrastive learning

After obtaining the low-dimensional representations of genes, we further optimize these representations using graph contrastive learning. To be specific, we use an inter-view contrastive loss to maximize the agreement of the same gene and distinguish from other genes in different views. Our objective is to learn high-quality gene representations, even in scenarios where there is limited prior knowledge about gene regulatory interactions. Therefore, we use the following strategy to select positive and negative samples. For any anchor gene *i* in the preserved graph, we contrast its representation $h_{i}^{\left(2\right)}$ with the representations of all genes in the perturbed graph except for gene *i*, denoted as ${\hat{h}}_{j}^{\left(2\right)}$. So the positive sample for the preserved graph can be set as $\left(h_{i}^{\left(2\right)},{\hat{h}}_{i}^{\left(2\right)}\right)$ and the negative samples can be set as $\left(h_{i}^{\left(2\right)},{\hat{h}}_{j}^{\left(2\right)}\right)$. In the perturbed graph, we adopt the same approach to construct positive and negative samples. The contrastive loss we used is formulated as follows:

(9) $$L_{\mathrm{con}}^{\mathrm{preser}}(h_{i}^{\left(2\right)})=-\log\frac{e^{\theta (h_{i}^{\left(2\right)},{\hat{h}}_{i}^{\left(2\right)})/\tau }}{\sum_{j=1}^{N}e^{\theta (h_{i}^{\left(2\right)},{\hat{h}}_{j}^{\left(2\right)})/\tau }},$$

 

(10) $$L_{\mathrm{con}}^{\mathrm{pertur}}({\hat{h}}_{i}^{\left(2\right)})=-\log\frac{e^{\theta ({\hat{h}}_{i}^{\left(2\right)},h_{i}^{\left(2\right)})/\tau }}{\sum_{j=1}^{N}e^{\theta ({\hat{h}}_{i}^{\left(2\right)},h_{j}^{\left(2\right)})/\tau }},$$

 

(11) $$L_{\mathrm{con}}=\frac{1}{2N}\sum_{i=1}^{N}[L_{\mathrm{con}}^{\mathrm{preser}}(h_{i}^{\left(2\right)})+L_{\mathrm{con}}^{\mathrm{pertur}}({\hat{h}}_{i}^{\left(2\right)})],$$

where *N* denotes the number of nodes and *τ* refers to the temperature parameter. θ(·,·) is the function that calculates the cosine similarity of these representations.

### 2.5 Link prediction

To facilitate the inference process, it is necessary to further acquire low-dimensional embeddings of genes. To further obtain low-dimensional embeddings of gene *i* of two views, we feed $h_{i}^{\left(2\right)}$ and ${\hat{h}}_{i}^{\left(2\right)}$ into MLPs:

(12) $$h_{i}=\text{MLP}(h_{i}^{\left(2\right)}),$$

 

(13) $${\hat{h}}_{i}=\hat{\text{MLP}}({\hat{h}}_{i}^{\left(2\right)}),$$

To infer the presence of a link between gene *i* and gene *j*. We can use a similar process to obtain low-dimensional embeddings $h_{j}$ and ${\hat{h}}_{j}$ for gene *j*.

Afterward, dot product operations are applied to the embeddings of gene *i* and gene *j* to compute the linkage scores between them in two different views. Subsequently, These scores are then transformed using a sigmoid function, which maps them to a probability value between 0 and 1:

(14) $$p=\text{sigmoid}(h_{i}^{T}\cdot h_{j}),$$

 

(15) $$\hat{p}=\text{sigmoid}({\hat{h}}_{i}^{T}\cdot {\hat{h}}_{j}).$$

We use a link prediction objective to optimize the model for predicting potential links between genes that have not been observed. Using predicted probability values from two different views, we assess the disparity between these predictions and the ground truth to optimize the model. In this work, we utilize binary cross-entropy as the loss function. The losses from the two perspectives are computed separately as follows:

(16) $$L_{\mathrm{preser}}=-\sum_{q=1}^{Q}y_{q}\;\text{log}(p)+\left(1-y_{q}\right)\;\text{log}(1-p),$$

 

(17) $$L_{\mathrm{pertur}}=-\sum_{q=1}^{Q}y_{q}\;\text{log}(\hat{p})+\left(1-y_{q}\right)\;\text{log}(1-\hat{p}),$$

where *y_q_* represents the label of the *q*th gene pair and *Q* refers to the number of gene pairs in the training set. It is noteworthy that when calculating the *L_pertur_*, we still utilize *y_q_* as the label because our goal is to reconstruct the original graph from the perturbed graph. To facilitate effective graph contrastive learning, we also incorporate contrastive loss. By combining the contrastive loss with the cross-entropy loss, we can obtain the final loss function of the model:

(18) $$L=L_{\mathrm{preser}}+L_{\mathrm{pertur}}+\beta L_{\mathrm{Con}}$$

where *β* is the hyper-parameter that controls the balance between the contrastive loss and the cross-entropy loss. In this work, we set the value of *β* to 0.5.

### 2.6 Datasets

We collect scRNA-seq datasets of seven cell types from BEELINE (Pratapa *et al.* 2020) to evaluate the performance of our GCLink model in predicting regulatory interactions between TFs and target genes. Specifically, the seven datasets include five mouse cell types: (i) mouse embryonic stem cells [mESC (Hayashi *et al.* 2018)]; (ii) mouse hematopoietic stem cells with erythroid-lineage (mHSC-E); (iii) mouse hematopoietic stem cells with granulocyte-monocyte-lineage (mHSC-GM); (iv) mouse hematopoietic stem cells with lymphoid-lineage (mHSC-L); (v) mouse dendritic cells [mDC (Shalek *et al.* 2014)], and two human cell types: (i) human embryonic stem cells [hESC (Kc *et al.* 2019)]; (ii) human mature hepatocytes [hHEP (Camp *et al.* 2017)]. These scRNA-seq datasets are accessible from Gene Expression Omnibus under the following accession numbers: GSE98664 (mESC), GSE81682 [mHSC (Nestorowa *et al.* 2016)], GSE48968 (mDC), GSE75748 (hESC), and GSE81252 (hHEP). Each dataset includes three ground truth networks derived from three different sources: (i) cell-type-specific chromatin immunoprecipitation sequencing [ChIP-seq (Moore *et al.* 2020)]; (ii) nonspecific ChIP-seq (Garcia-Alonso *et al.* 2019); (iii) functional interaction networks documented in STRING (Szklarczyk *et al.* 2019) database. The details of each dataset and their respective ground truth networks are summarized in Table 1.

**Table 1.** The details about seven scRNA-seq datasets.^a^

Cell types	Cells	Cell-type-specific			Nonspecific			STRING		
		TFs	Genes	Density	TFs	Genes	Density	TFs	Genes	Density
mHSC-E	1071	29 (33)	691 (1177)	0.578 (0.566)	144 (147)	442 (674)	0.022 (0.020)	156 (161)	291 (413)	0.029 (0.027)
mHSC-GM	889	22 (23)	618 (1089)	0.543 (0.565)	82 (88)	297 (526)	0.030 (0.029)	92 (100)	201 (344)	0.040 (0.037)
mHSC-L	847	16 (16)	525 (640)	0.525 (0.507)	35 (37)	164 (192)	0.048 (0.043)	39 (40)	70 (81)	0.048 (0.045)
mESC	421	88 (89)	977 (1385)	0.345 (0.347)	516 (522)	890 (1214)	0.015 (0.013)	495 (499)	638 (785)	0.024 (0.021)
hESC	758	34 (34)	815 (1260)	0.164 (0.165)	283 (292)	753 (1138)	0.016 (0.014)	343 (351)	511 (695)	0.024 (0.021)
hHEP	425	30 (31)	874 (1331)	0.379 (0.377)	322 (332)	825 (1217)	0.015 (0.013)	409 (414)	646 (874)	0.028 (0.024)
mDC	383	20 (21)	443 (684)	0.085 (0.082)	250 (254)	634 (969)	0.019 (0.016)	264 (273)	479 (664)	0.038 (0.032)

a Each dataset contains three ground truth networks (cell-type-specific, nonspecific, and STRING). The values outside the parentheses represent details for all significantly varying TFs and the 500 most-varying genes (TFs+500), while the values inside the parentheses represent the details for all significantly varying TFs and the 1000 most-varying genes (TFs+1000).

For all scRNA-seq datasets, we follow the same data preprocessing steps as BEELINE. Initially, genes expressed in <10% of cells are filtered out. Subsequently, the Bonferroni correction is applied, and genes with corrected *P*-values higher than 0.01 are excluded. Finally, we adopt the variance ranking strategy in BEELINE to select the top 500 and 1000 highly variable genes, resulting in the generation of 14 distinct datasets.

For all ground truth networks, observed gene regulatory interactions are treated as positive samples. Due to varying network densities across datasets, we use two distinct approaches to select negative samples.

For the STRING and nonspecific ChIP-seq networks, we first partition the positive samples into a positive training set (comprising 2/3 of the positive samples) and a positive test set (comprising 1/3 of the positive samples). Then, we extract 1/5 of the samples from the positive training set to form the positive validation set. Negative training and negative validation sets are then created by uniformly and randomly sampling from unobserved interactions, maintaining a 1:1 balance between positive and negative samples. In the test set, given the low network density, we maintain a constant ratio between positive and negative samples. Further details on the negative sample sampling strategy are presented in Supplementary Section SC.

For the cell-type-specific ChIP-seq ground truth networks, due to the significantly higher degrees of TFs, we sample 2/3 of the positive samples for training, 1/10 for validation, and the remainder for testing. Notably, during model training, we use only the samples from the training set to construct the adjacency matrix, thereby preventing information leakage. Details of the training, validation, and testing sets for the three types of ground truth networks are provided in Supplementary Table S1.

## 3 Results

### 3.1 Experimental settings

**Figure 1.**
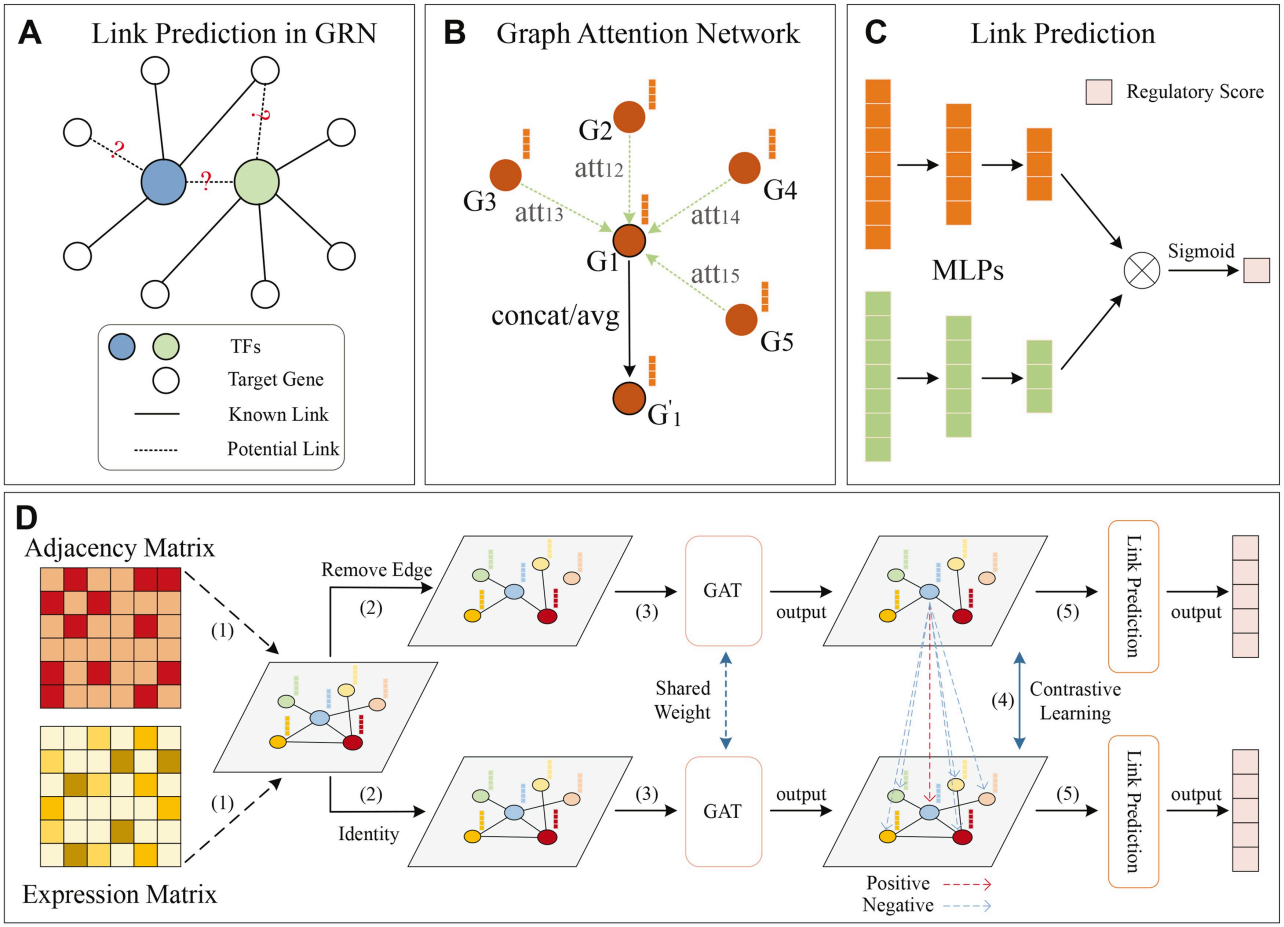
Overview of GCLink framework. (A) We formulate the GRN inference problem as a link prediction task, which aims to predict potential links based on known links. (B) The information aggregation in the GAT layer. The aggregated features from each gene are concatenated or averaged to obtain the updated gene feature. (C) The description of the link prediction module. The gene representations are fed into MLPs to obtain low-dimensional embeddings. The regulatory scores between genes are computed by taking the dot product of their embeddings and passing the result through a sigmoid function. (D) The pipeline of GCLink. (1) Initially, the input for GCLink consists of a gene expression matrix and an adjacency matrix, which indicates the observed gene regulatory interactions in the training set. We treat gene expression values as node features and construct the original graph using the adjacency matrix. (2) Next, two graph views are generated from the original graph using random edge removal and preservation methods, respectively. (3) Then, we extract gene representations through GAT layers. (4) Subsequently, we apply graph contrastive learning to enhance gene representation learning, maximizing the agreement of positive gene pairs. (5) Finally, we use the link prediction module to predict the regulatory scores of gene pairs.

To expedite model convergence, we use a pre-training strategy before each training, conducted in a supervised manner. Notably, contrastive learning is omitted during pre-training. Nevertheless, we maintain the graph augmentation procedure described in Fig. 1. We optimized our model using the Adam optimizer, with an initial learning rate of 0.003, training for 20 epochs. In most cases, a smaller learning rate tends to yield optimal performance, and there is no need to set a large number of epochs as the model typically converges quickly. For graph augmentation, we set the probability of randomly removing edges to 0.2 by default. The choice of this hyperparameter will be discussed in subsequent experiments.

### 3.2 Performance on benchmark datasets

In order to assess the superiority of our GCLink model, we conducted a comparative analysis with six baseline methods. These methods include three supervised learning methods, i.e. GENELink (Chen and Liu 2022), CNNC (Yuan and Bar-Joseph 2019), GNE (Kc *et al.* 2019), and three unsupervised learning methods, namely DeepSEM (Shu *et al.* 2021), GENIE3 (Huynh-Thu *et al.* 2010), and GRNBoost2 (Moerman *et al.* 2019). To maintain fairness, supervised learning methods were evaluated using identical training sets. Evaluation metrics included AUROC (Area Under the Receiver Operating Characteristic curve) and AUPRC (Area Under the Precision-Recall curve), with consistent evaluation methodology applied across all methods and an identical test set utilized.

**Figure 2.**
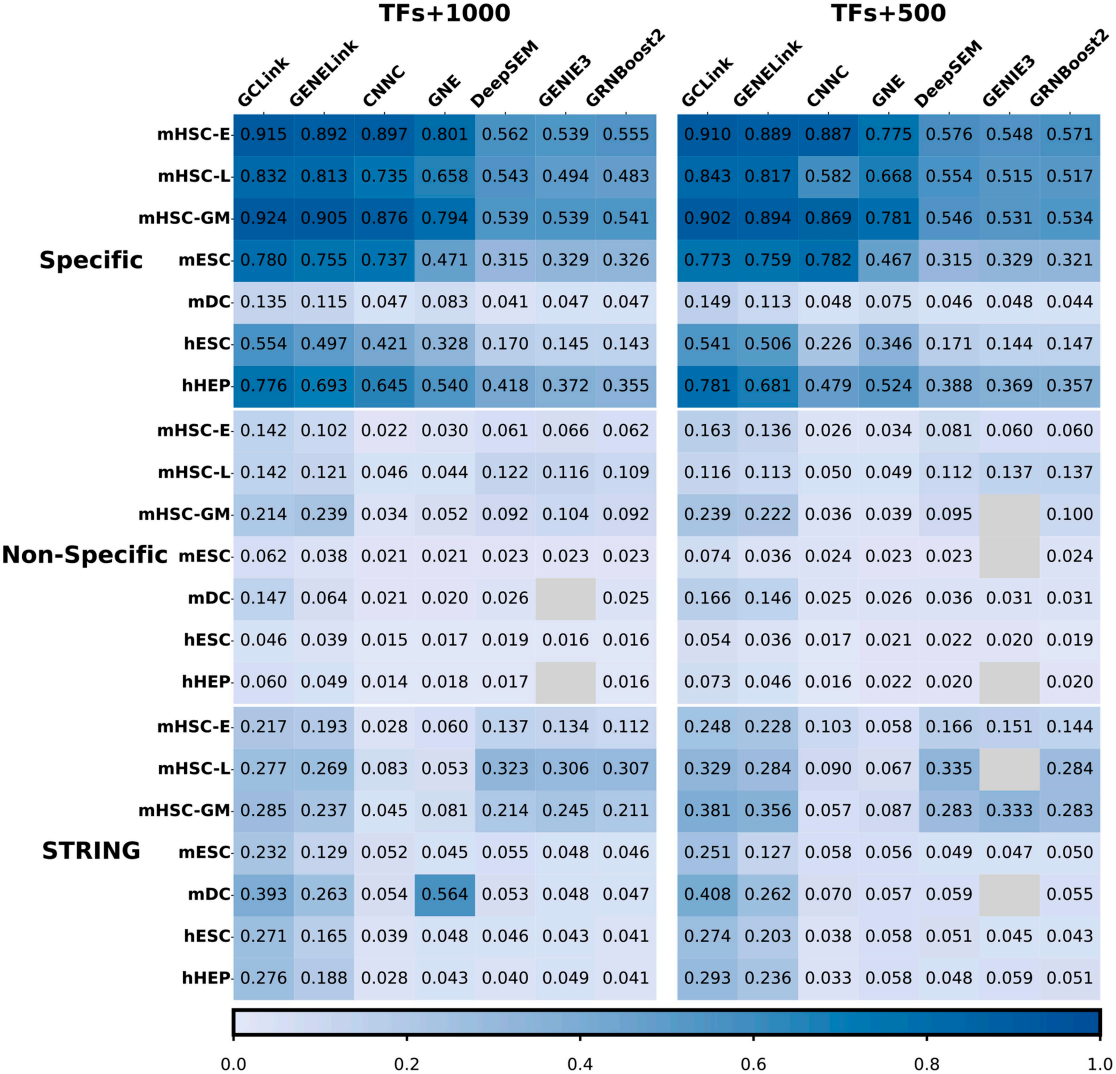
Heatmap depicting AUPRC scores of various methods evaluated on benchmark datasets. The left column shows the AUPRC scores for datasets with the top 1000 most-varying genes, while the right column displays the AUPRC scores for datasets with the top 500 most-varying genes. Colors deepen with higher values, while masked values indicate missing values.

Firstly, we conducted a comparative analysis between GCLink and unsupervised learning methods. Figure 2 and Supplementary Fig. S1 demonstrates the superior performance of GCLink across almost all datasets in terms of AUROC and AUPRC scores. It’s noteworthy that GCLink and other supervised learning methods showed inferior performance compared to unsupervised learning methods on only one dataset. Further investigation revealed that this dataset had an exceptionally low number of edges in its training set. This observation indicates the significant impact of excessively sparse networks on the performance of supervised learning models.

Then we conducted a comparative evaluation of GCLink with two supervised learning methods, namely CNNC and GNE. As depicted in Fig. 2 and Supplementary Fig. S1, although CNNC and GNE demonstrate commendable performance, GCLink consistently outperforms both CNNC and GNE in terms of AUROC and AUPRC scores across all datasets. This finding reinforces the superiority of GNN over CNN in accurately inferring GRNs.

Finally, we compared GCLink with the state-of-the-art GNN-based method, GENELink, to illustrate the efficacy of contrastive learning in accurately distinguishing positive gene pairs from negative ones. Based on the insights derived from Fig. 2 and Supplementary Fig. S1, GCLink demonstrates superior performance to GENELink in both AUROC and AUPRC scores on cell-type specific ChIP-seq ground truth networks. In contrast, on nonspecific ChIP-seq ground truth networks, GCLink demonstrates comparable AUROC performance to GENELink, while outperforming GENELink on 64.3% (9/14) of the datasets and achieves a higher AUPRC than GENELink on 85.7% (12/14) of the datasets. Furthermore, GCLink also demonstrated promising performance on STRING ground truth networks, surpassing GENELink in terms of AUROC on 64.3% (9/14) of the datasets and achieving superior AUPRC across all datasets compared to GENELink.

In addition, the inference performance of GCLink and other supervised methods consistently declined as the density of the prior network decreased. This is due to the reliance of supervised learning methods, particularly those based on GNNs, on labeled data. However, GCLink outperforms both unsupervised learning methods and other supervised learning methods on most (24/28) datasets with extremely low network densities in terms of AUPRC, suggesting that GCLink has a higher tolerance for sparse networks.

These results clearly demonstrate that integrating contrastive learning enables the model to discern subtle differences between positive and negative gene pairs, thereby improving the precision of gene representation learning.

### 3.3 Few-shot studies

Note that we utilized random edge removal for graph augmentation, which effectively enhanced the model’s ability to infer potential edges in sparse networks. Moreover, the incorporation of contrastive learning further improved the model’s capacity to learn the representation of genes. Notably, our model consistently exhibited exceptional performance even when subjected to random edge removal in our experiments. This observation suggests that our model can capture more essential gene features which are expected to facilitate the inference of GRNs.

Due to the extremely limited number of known gene regulatory interactions, there may be either no known gene regulatory interactions or only a few known regulatory interactions for specific cell types or cellular states. To test the generalization performance of our model, we consider a scenario where there are few known regulatory interactions for the target cell line (referred to as a few-shot scenario). In this case, we pretrain the model using known gene regulatory interactions from other cell lines and subsequently fine-tune it with a small amount of known regulatory interactions from the target cell line. Specifically, we selected a cell line with a substantial number of known gene regulatory interactions as the source cell line to pretrain the model. For the target cell line, we performed stratified sampling to select 5% of the known regulatory interactions for fine-tuning, with the remaining data used as the test set to evaluate model performance.

To facilitate knowledge transfer between different cell lines for the model, we need to ensure consistent input feature dimensions. To achieve this, we performed singular value decomposition on the feature matrices of both the source and target cell lines before feeding them into the model. Then we maintained consistency in the pretraining process for the source cell line and the fine-tuning process for the target cell line, with the only distinction being a reduction in the number of epochs during fine-tuning. It is worth noting that when pretraining the model using the source cell line, we utilized all available labeled data. When fine-tuning the model on the target cell line, we used 5% of the labeled data for fine-tuning and evaluated the model’s performance on the remaining 95% labeled data, using AUROC and AUPRC as metrics to assess the model’s performance.

**Figure 3.**
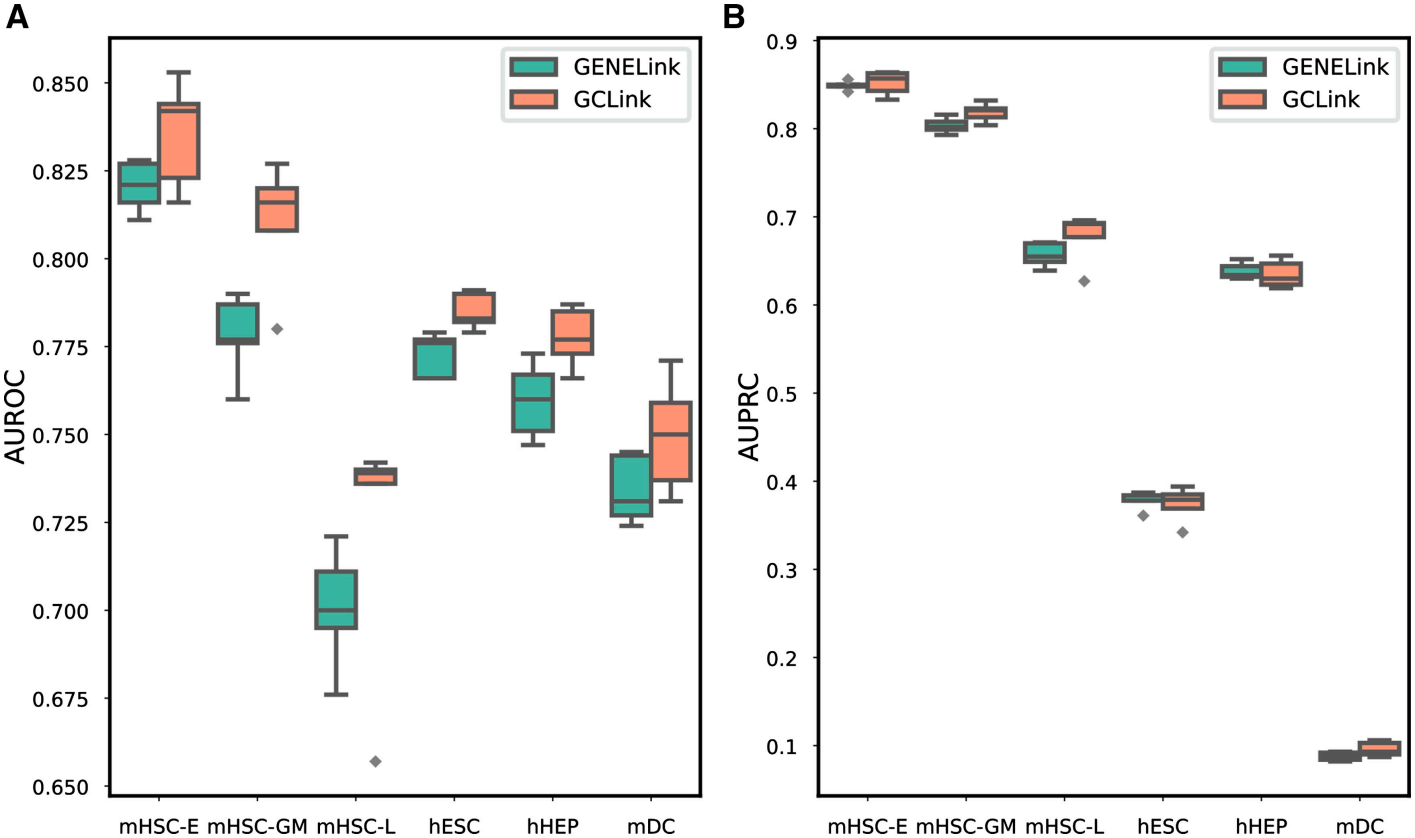
Few-shot experimental results of GCLink and GENELink, evaluated on various target cell lines, in terms of (A) AUROC and (B) AUPRC. Both GCLink and GENELink pretrain on the mESC (TFs+1000) dataset, using the cell-type-specific network as the source dataset, and then fine-tune on the target cell lines.

**Figure 4.**
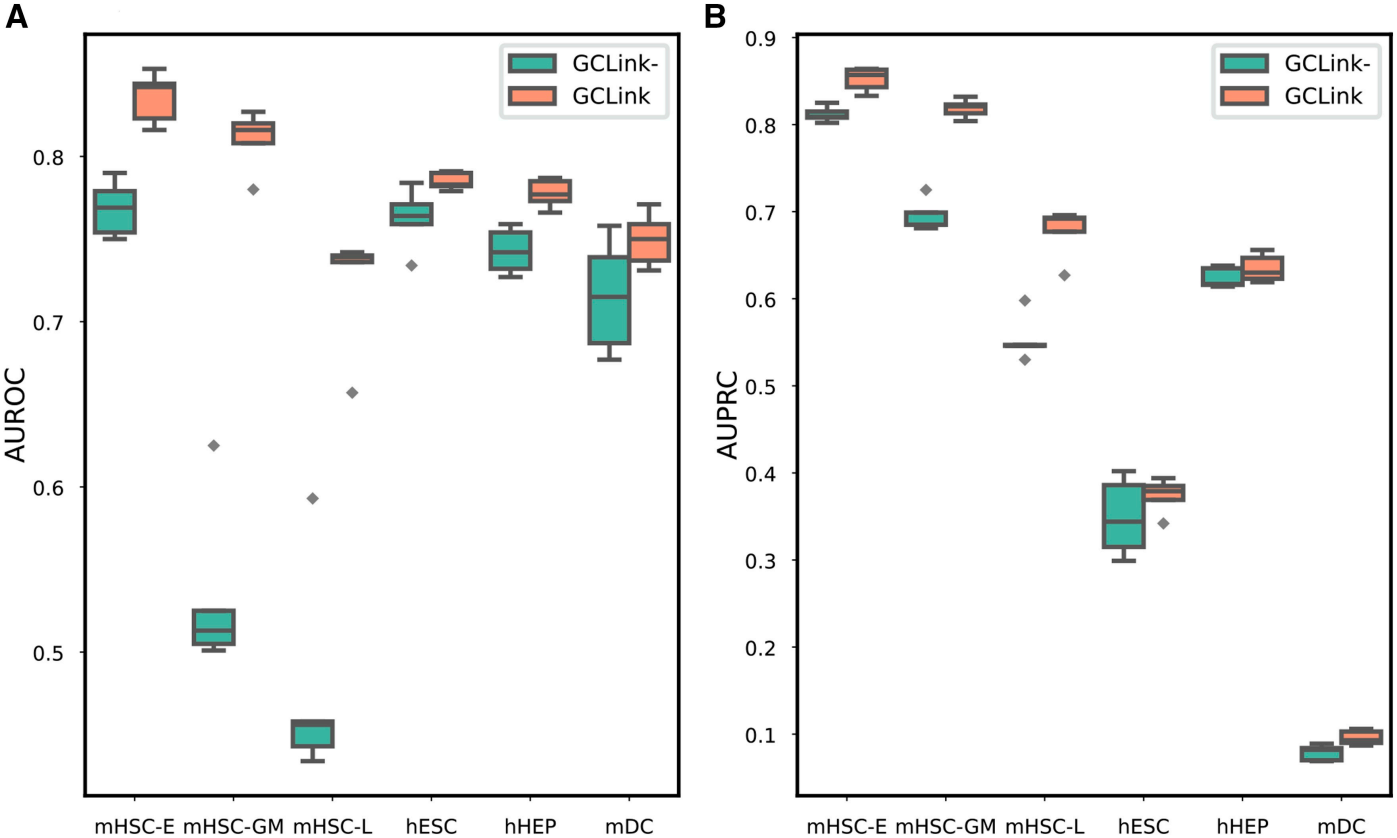
Few-shot experimental results of GCLink and GCLink-, evaluated on various target cell lines, in terms of (A) AUROC and (B) AUPRC. GCLink- denotes the model trained directly on the target cell line without pretraining.

Considering that the size of the source cell line used for pretraining needs to be much larger than that of the target cell line, we selected the mESC as the source cell line, while the remaining cell lines were evaluated individually as the target cell lines. As shown in Fig. 3, our model outperforms GENELink in terms of AUROC and AUPRC, indicating that our GCLink model exhibits stronger transferability in few-shot scenarios. Furthermore, to validate the benefit of pretraining the model on the source cell line, we compared the performance of training our model directly on the target cell line with the performance of pretraining on the source cell line followed by fine-tuning on the target cell line. As shown in Fig. 4, pretraining on the source cell line followed by fine-tuning on the target cell line can achieve better results than directly training on the target cell line. We can also find from Fig. 4 that the performance of training directly on the target cell line is greatly affected when the size of the training data in the target cell line is very small, such as mHSC-GM and mHSC-L cell lines. The above results demonstrate that GCLink possesses remarkable transferability and can adapt to sparse GRNs.

### 3.4 Hyperparameter analysis

The performance of our model can be influenced by various hyperparameters, such as learning rate, number of epochs, and others. However, in this study, the most crucial and analytically significant parameter is the probability of randomly removing edges. Selecting an appropriate probability can significantly enhance the model’s performance and generalization ability. Nevertheless, the optimal probability often varies depending on the scale of the dataset. To address this, we conducted experiments to analyze the selection criteria for this hyperparameter.

**Figure 5.**
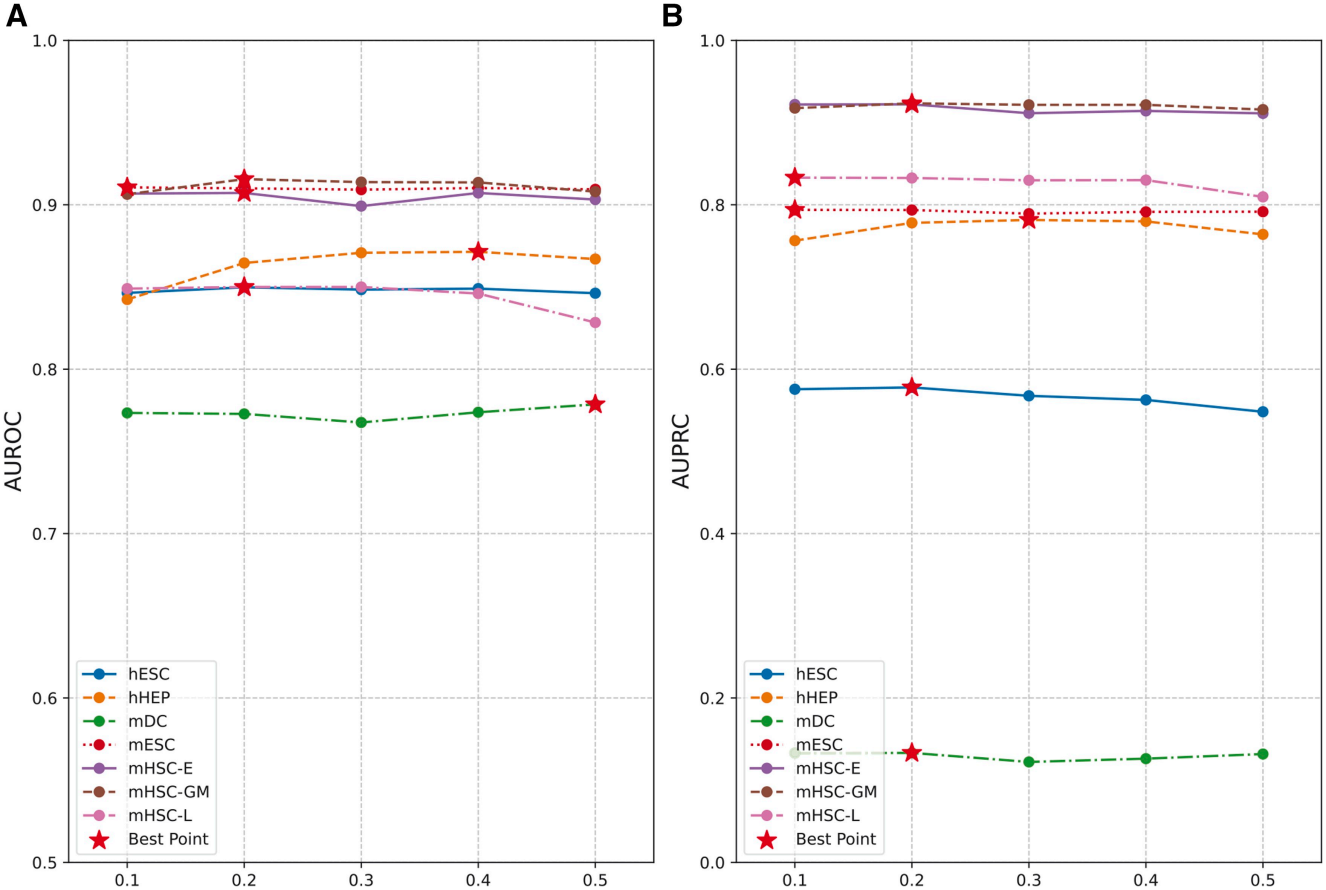
The experimental results of GCLink trained with different probabilities of randomly removing edges in terms of (A) AUROC and (B) AUPRC. All of them are evaluated on the cell-type-specific networks (TFs+1000).

As shown in Fig. 5, we conducted experiments on seven different cell types. For both AUROC and AUPRC, setting the edge removal probability to 0.2 resulted in the highest number of optimal performances. Moreover, using a smaller probability often yielded more stable and effective outcomes compared to a larger probability. This is because a high edge removal probability may lead to excessive loss of information, while a smaller probability enhances the model’s generalization ability in sparse scenarios without negatively impacting its performance.

### 3.5 Ablation studies

In this study, we use graph contrastive learning to enhance the model’s feature extraction capability. To validate the effectiveness of contrastive learning, we conducted ablation experiments across seven datasets.

**Figure 6.**
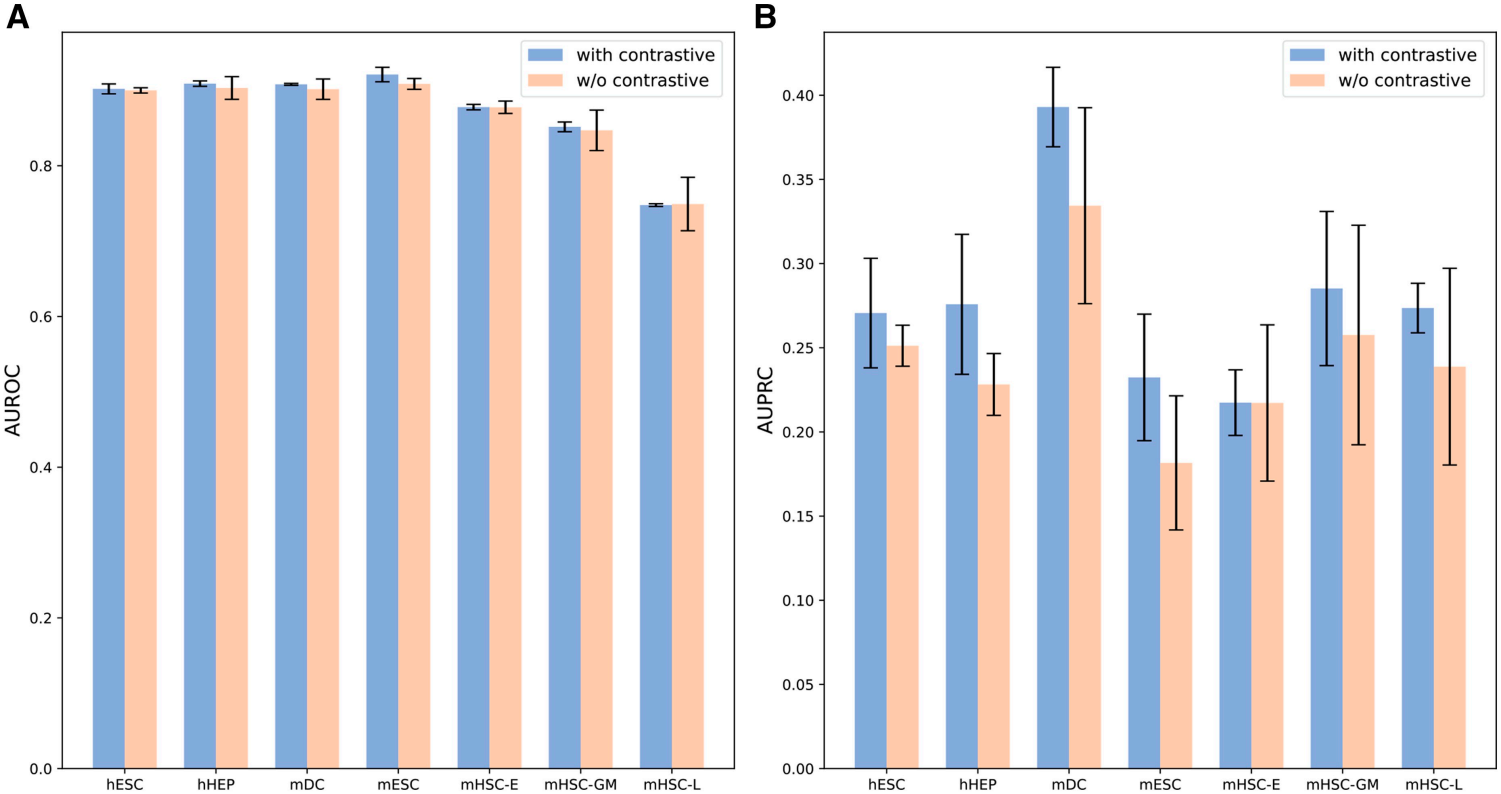
The experimental results of GCLink trained with and without contrastive learning, in terms of (A) AUROC and (B) AUPRC. All of them are evaluated on the STRING networks (TFs+1000).

By removing the contrastive learning component in the experiments and comparing the means and standard deviation with the control group, we demonstrate the effectiveness of contrastive learning. The experimental results shown in Fig. 6 reveal that that contrastive learning effectively reduces the standard deviation in AUROC and improves the model’s performance in terms of AUPRC.

### 3.6 Sensitivity studies

GCLink relies on GAT to capture potential interactions between genes, while GAT requires a observed network to perform message passing. Consequently, we further investigate the performance of GCLink in the presence of noise in the observed network. To do this, we perturbed the observed network by randomly flipping edges with a certain probability, i.e. randomly removing existing edges and adding new ones.

**Figure 7.**
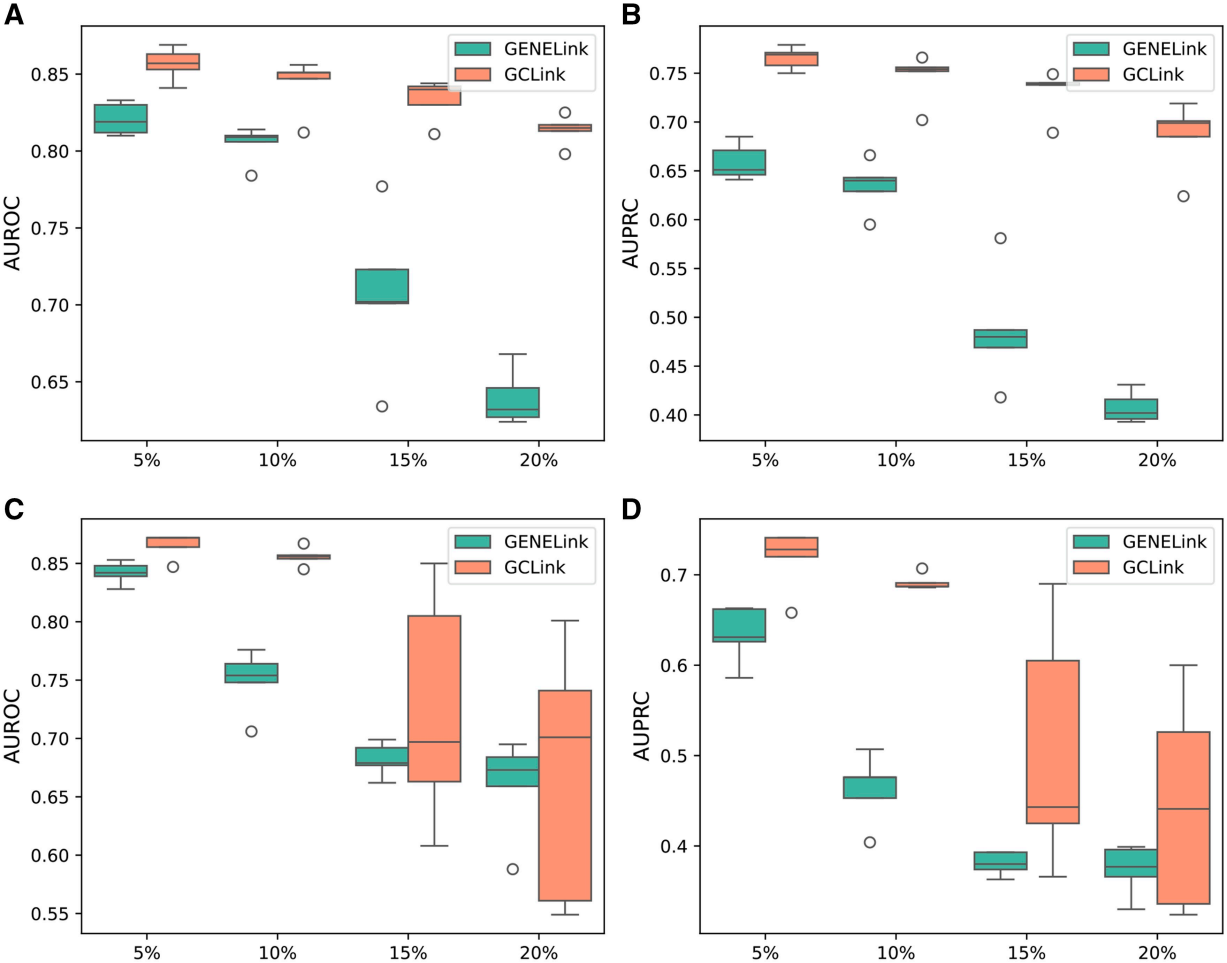
The experimental results of GCLink and GENELink evaluated on the hHEP and mESC datasets with varying levels of network noise, in terms of (A) AUROC on hHEP, (B) AUPRC on hHEP, (C) AUROC on mESC, and (D) AUPRC on mESC. All of them are evaluated on the cell-type-specific networks (TFs+1000).

We conducted experiments using GCLink and GENELink across multiple datasets. We observed that as noise levels increased, both GCLink and GENELink exhibited a gradual decline in performance. However, on most datasets, GCLink was less affected by noise compared to GENELink. As shown in Fig. 7 and Supplementary Fig. S3, on most datasets, GCLink outperformed GENELink in terms of both AUROC and AUPRC, demonstrating its higher tolerance to noise.

### 3.7 Case studies

In this work, we formulate the GRN inference problem as a link prediction task, where the model’s ability to predict potential interactions is of critical importance. We apply our model to the hESC dataset (with the cell-type specific ground truth network as the training set) and evaluate the performance of our model in inferring potential regulatory interactions.

Table 2 shows the top 10 gene pairs with the highest prediction scores. We found evidence from existing studies supporting regulatory interactions for eight of these pairs. We also examined the top 10 gene pairs with the highest predicted scores by GENELink, as presented in Supplementary Table S2. Therefore, the remaining two gene pairs that could not be validated are more likely to represent true regulatory interactions. Furthermore, we extracted the top 100 predicted potential gene pairs and visualized the inferred GRN, as depicted in Supplementary Fig. S2. In summary, GCLink has shown the capacity to accurately infer known gene regulatory interactions and effectively predict potential regulatory interactions. This capability holds great promise in assisting researchers in discovering novel gene regulatory interactions.

**Table 2.** Top-10 gene pairs predicted by GCLink on hESC dataset.

TF	Target gene	Reference
TFAP2A	MORC4	ChIPBase (Huang *et al.* 2023)
TFAP2A	ST8SIA1	Harmonizome (Rouillard *et al.* 2016)
TFAP2A	POLB	Harmonizome (Rouillard *et al.* 2016)
TFAP2A	DYNC2LI1	ChIPBase (Huang *et al.* 2023)
TFAP2A	RASGRP3	
TFAP2A	POLR2G	
TFAP2A	TCN2	ChIPBase (Huang *et al.* 2023)
TFAP2A	DMBX1	ChIPBase (Huang *et al.* 2023)
TFAP2A	HOOK1	ChIPBase (Huang *et al.* 2023)
TFAP2A	BTBD10	ChIPBase (Huang *et al.* 2023)

## 4 Discussion

The accumulation of scRNA-seq data has advanced the inference of gene regulatory interactions within cells. However, the high dimensionality, noise, and dropout rates associated with scRNA-seq data also pose challenges for traditional methods in inferring GRNs (GRNs). Moreover, existing GRN inference methods still suffer from issues such as time-consuming computations and excessive reliance on labeled data. In this work, we propose GCLink aimed at reducing the dependency on labeled data and enhancing the accuracy of GRN inference.

GCLink leverages scRNA-seq data and the limited known regulatory interactions between TFs and target genes to infer GRNs. By treating TFs and genes as nodes and the regulatory interactions between TFs and target genes as edges, we formulate GRN inference as a link prediction problem. To capture the influence of neighboring genes when inferring regulatory interactions between TFs and target genes, we use Graph Attention Networks (GAT) to aggregate information from neighboring genes. Furthermore, we use graph augmentations and introduce a contrastive learning strategy to enhance the learning of gene representations from sparse regulatory interactions. Finally, we utilize MLPs to extract low-dimensional gene embeddings and use dot product operations for link prediction. We evaluate the performance of GCLink on 14 benchmark datasets and compare it with other baseline methods. The experimental results show that GCLink consistently outperforms other methods on most datasets. We also conduct few-shot experiments to assess the performance of our model with limited known regulatory interactions. Experimental results demonstrate that our GCLink exhibits good generalization performance even when only a few labeled data are available. By applying our GCLink to the hESC dataset, we also infer some novel regulatory interactions between TFs and target genes successfully.

However, supervised GNNs require high-quality gold standard gene expression data and networks, as GNN-based models rely on these networks for message passing and feature aggregation. The results of sensitivity studies show that when the observed network contains significant noise, both our model and GENELink experience decreased performance. Additionally, determining the optimal hyperparameters is a crucial issue. As demonstrated by the hyperparameter analysis experiments, the optimal hyperparameters often vary across different datasets. Therefore, further discussion can be conducted on methods for selecting the random edge removal probability based on the dataset scale.

In future work, we aim to further improve the transferability of our model, enabling it to achieve robust performance even in fully unsupervised scenarios. This includes exploring few-shot or zero-shot learning approaches to adapt the model effectively. Furthermore, we will focus on refining graph augmentation methods to improve the model’s stability, which is crucial for inferring unknown gene regulatory interactions.

## Supplementary Material

btaf074_Supplementary_Data

## Data Availability

The source code and data are available at https://github.com/Yoyiming/GCLink.
