# Supplementary material for: GCLink: a graph contrastive link prediction framework for gene regulatory network inference
Source: Bioinformatics. 2025 Feb 17;41(3):btaf074. doi: 10.1093/bioinformatics/btaf074 (PMC11881698; doi:10.1093/bioinformatics/btaf074)
Supplement: btaf074_Supplementary_Data [file btaf074_supplementary_data.pdf]

A. Details of GCLink

In GCLink, we employed two layers of Graph Attention Networks (GAT) to extract features of genes, with each GAT layer comprising three attention heads. For the first GAT layer, we utilized a concatenation approach to integrate the features from each attention head, with an output feature dimension of 128 for each attention head, resulting in a total feature dimension of 384 for the first GAT layer. In the second GAT layer, we employed a mean aggregation method to combine the features from each attention head, with an output feature dimension of 64 for each attention head, resulting in an output feature dimension of 64 for the second GAT layer. In the two channels of MLPs, we compressed the 64-dimensional features outputted by GAT through two linear layers, reducing the gene features in a 64-32-16 manner, ultimately obtaining a 16-dimensional gene representation. In the GAT layers, we employed the ELU nonlinearity as the activation function, while in the MLP layers, we used the LeakyReLU nonlinearity as the activation function.

The time complexity of GCLink is primarily determined by the GAT layers. For a single GAT attention head, the time complexity can be expressed as $O(|V|\,FF^{'}+|E|\,F^{'})$ (Velickovic et al., 2017), where $|V|$ refers to the number of genes and $|E|$ refers to the number of observed TF-target interactions. F and $F^{'}$ represent the dimensions of the input features and the computed features, respectively. For multi-head attention, the storage and parameter requirements will be multiplied by a factor of K, where K is the number of attention heads. However, since the calculation for each head is independent and can be parallelized, the time complexity remains consistent with that of a single attention head (Velickovic et al., 2017). From the above time complexity analysis, it can be seen that the time complexity of GCLink primarily depends on the number of genes, the number of cells, and the quantity of interactions. Since the number of cells is typically much smaller than the number of genes, and GRNs are usually sparse, the time complexity is almost linear with the number of genes. In such cases, current GPUs are generally able to efficiently process a large number of genes, for example, 10,000 genes or even more. In this study, we employed the NVIDIA GeForce RTX 3090 GPU for our computations. Training our model on the mESC dataset (cell-type-specific network, TFs + 1000) using all observed interactions (a total of 42,795 edges) required 628.39 seconds and only utilized approximately 1/8 of the GPU memory.

B. The implementation of baselines

In this section, we will provide details of our implementation of the baseline models. For the implementation of the GENIE3 (Huynh-Thu et al., 2010) and GRNBoost2 (Moerman et al., 2019) methods, we utilized scripts provided by BEELINE (Pratapa et al., 2020) and used default parameters. In the case of DeepSEM (Shu et al., 2021), we followed the tutorials provided by authors to obtain the inferred weight matrices and selected the inference results of gene pairs in the test set for evaluation. Regarding CNNC (Yuan and Bar-Joseph, 2019), due to issues with convergence when using early stopping, we disabled early stopping to ensure more accurate evaluation results and set a fixed number of epochs to 60 to guarantee reasonable outcomes on all datasets. For GNE (Kc et al., 2019) and GENELink (Chen and Liu, 2022), we used the default parameters provided by the authors for our implementation.

C. The Negative Sample Sampling Strategy for the Benchmark Datasets

In this section, we will provide detailed strategies for negative sample selection. For the STRING and non-specific ChIP-seq networks, the prior network exhibits very low density, indicating a severe limitation in the number of positive samples. Sampling negative samples at a 1:1 ratio with positive samples would result in an overly small test set, leading to unreliable evaluation results. To address this, we ensure that the positive-to-negative sample ratio in the test set closely mirrors the ratio observed in the prior network, as detailed below:

$$\frac{Positive}{Negative}=\frac{Density}{1-Density}. \tag{1}$$

For the cell-type-specific networks, we employed a hard negative sampling strategy to introduce more discriminative information (Yang et al., 2022) and enhance gene representation learning. Specifically, for an observed gene pair (TF_i, TG_j), we sampled unobserved gene pairs (TF_i, TG_{nt}) as negative examples, where TF_i represents transcription factor (TF) i and TG_j refers to target gene j; TG_{nt} denotes non-target genes.

D. Supplementary Figures

		TFs+1000							TFs+500						
		GCLink	GENELink	CNNC	GNE	DeepSEM	GENIE3	GRNBoost2	GCLink	GENELink	CNNC	GNE	DeepSEM	GENIE3	GRNBoost2
Specific	mHSC-E	0.904	0.889	0.890	0.785	0.545		0.507	0.890	0.874	0.875	0.748	0.550		0.517
	mHSC-L	0.850	0.837	0.770	0.706	0.602	0.556	0.528	0.848	0.832	0.641	0.701	0.577	0.542	0.530
	mHSC-GM	0.914	0.901	0.879	0.790	0.553	0.527	0.509	0.899	0.893	0.873	0.786	0.566	0.525	0.517
	mESC	0.902	0.879	0.867	0.680	0.522	0.532	0.527	0.900	0.882	0.892	0.672	0.523	0.531	0.521
	mDC	0.782	0.718	0.529	0.687		0.522	0.514	0.773	0.708	0.538	0.629		0.502	
	hESC	0.840	0.834	0.771	0.695	0.576			0.849	0.835	0.637	0.725	0.575		0.504
	hHEP	0.874	0.834	0.786	0.713	0.556	0.539	0.507	0.867	0.829	0.622	0.696	0.548	0.535	0.512
Non-Specific	mHSC-E	0.746	0.750	0.516	0.591	0.576	0.569	0.592	0.751	0.750	0.507	0.585	0.606	0.571	0.585
	mHSC-L	0.779	0.773	0.514	0.511	0.582	0.607	0.580	0.551	0.580	0.506	0.510	0.585	0.591	0.587
	mHSC-GM	0.724	0.759		0.598	0.605	0.646	0.637	0.702	0.726	0.529	0.572	0.602		0.645
	mESC	0.803	0.761	0.603	0.590	0.568	0.561	0.547	0.795	0.740	0.594	0.589	0.550		0.545
	mDC	0.785	0.761	0.580	0.566	0.550		0.521	0.803	0.799	0.610	0.584	0.583	0.535	0.530
	hESC	0.703	0.705	0.531	0.542	0.542	0.516	0.505	0.713	0.685	0.531	0.566	0.549	0.517	0.509
	hHEP	0.730	0.728	0.519	0.571	0.528		0.509	0.718	0.713		0.584	0.531		0.517
STRING	mHSC-E	0.878	0.885		0.663	0.632	0.645	0.630	0.866	0.889		0.643	0.673	0.679	0.676
	mHSC-L	0.748	0.772	0.617	0.581	0.684	0.682	0.696	0.769	0.768	0.628	0.630	0.700		0.668
	mHSC-GM	0.852	0.865	0.523	0.640	0.675	0.725	0.695	0.866	0.874	0.560	0.671	0.729	0.773	0.714
	mESC	0.921	0.885	0.678	0.650	0.587	0.609	0.585	0.912	0.875	0.671	0.657	0.569	0.592	0.578
	mDC	0.908	0.892	0.639	0.636	0.546	0.550	0.537	0.906	0.878	0.677	0.598	0.586		0.547
	hESC	0.902	0.882	0.617	0.654	0.625	0.642	0.600	0.902	0.890	0.609	0.653	0.621	0.635	0.592
	hHEP	0.909	0.890	0.529	0.647	0.594	0.632	0.582	0.907	0.892	0.531	0.668	0.613	0.632	0.583

Color scale: 0.5 0.6 0.7 0.8 0.9 1.0

Fig. S1: Heatmap depicting AUROC scores of various methods on benchmark datasets. The left column shows the results for datasets with the top 1000 most-varying genes, while the right column displays results for datasets with the top 500 most-varying genes. Colors deepen with higher values, while masked values indicate either missing values or performance inferior to random predictors.

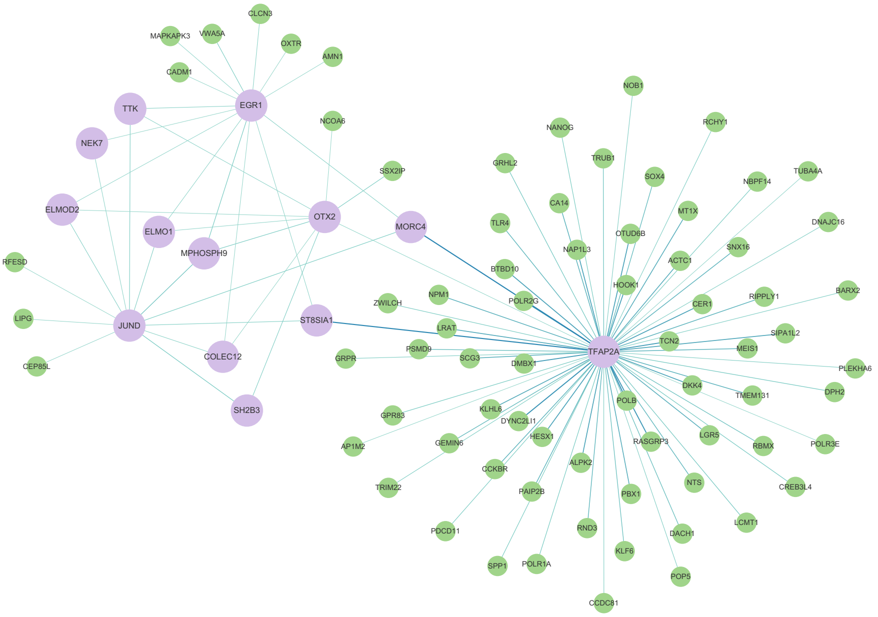

Fig. S2: Top-100 predicted potential gene regulatory interactions of the hESC (TFs+1000) dataset. The purple nodes represent TFs, while the green nodes denote target genes. The edge color intensity corresponds to the prediction score, with deeper and more opaque colors indicating higher scores.

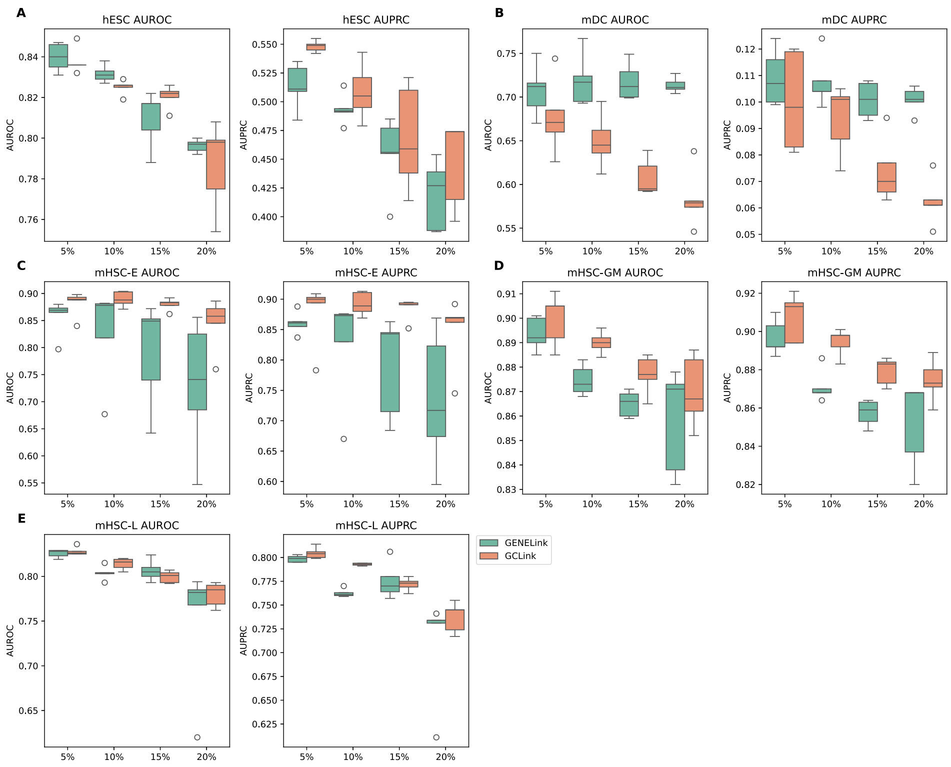

Fig. S3: The performance of GCLink and GENELink with varying levels of network noise on hESC, mDC, mHSC-E, mHSC-GM and mHSC-L datasets in terms of AUROC and AUPRC.

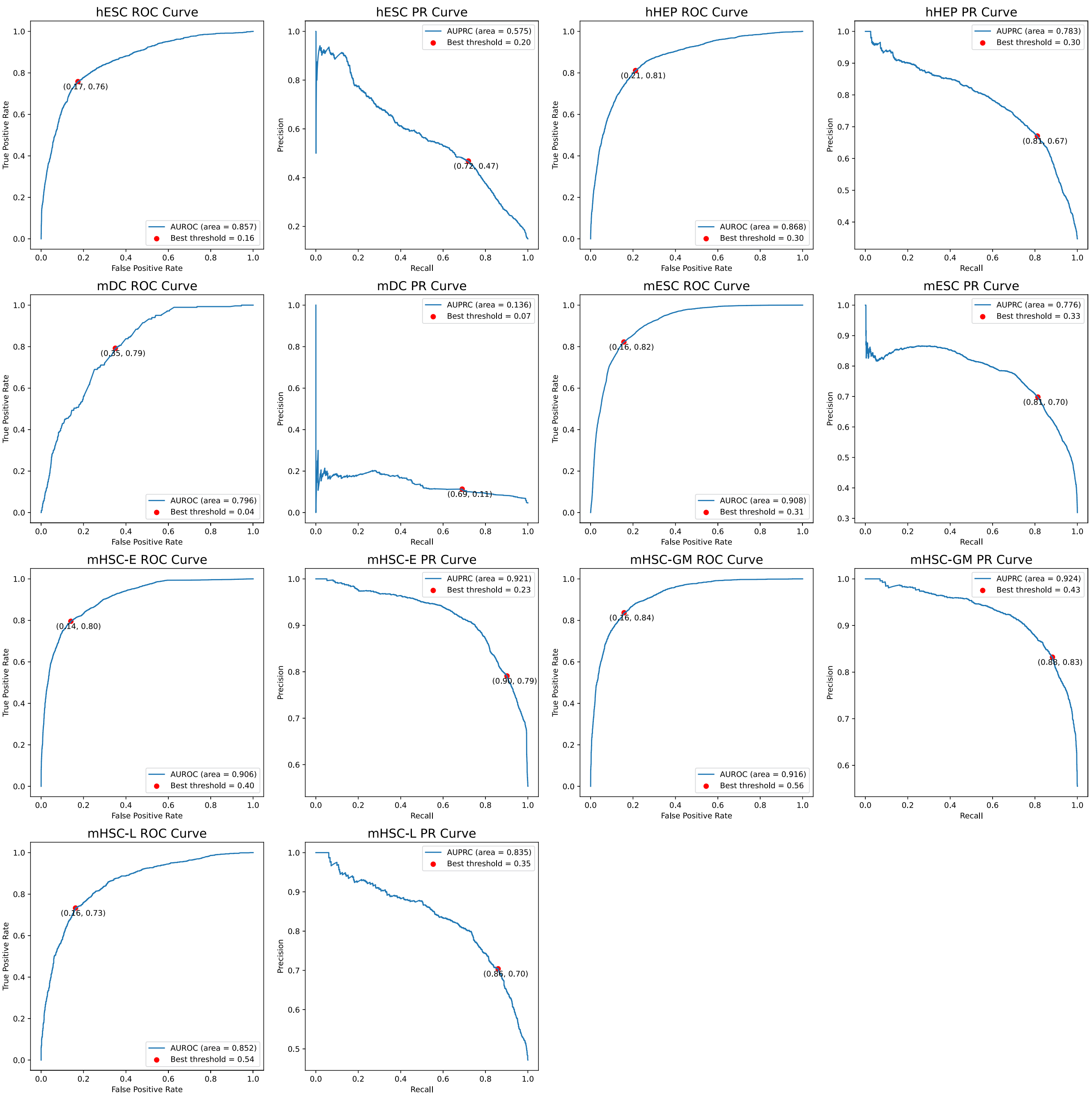

Fig. S4: The ROC curves and PR curves of seven cell type datasets (cell-type-specific network, TFs+1000).

E. Supplementary Tables

Table S1. The number of edges in seven single-cell RNA-sequencing datasets. Each dataset contains three ground truth networks. Values outside the parentheses represent the number of edges for all significantly varying TFs and the 500 most-varying genes (TFs+500), while values inside the parentheses represent the number of edges for all significantly varying TFs and the 1000 most-varying genes (TFs+1000).

Cell type	Cell-type-specific			Non-Specific			STRING		
	Train	Validation	Test	Train	Validation	Test	Train	Validation	Test
mHSC-E	7731(14707)	1154(2197)	2672(5071)	774(1064)	134(204)	517(692)	759(1003)	118(174)	494(649)
mHSC-GM	4925(9460)	733(1414)	1706(3261)	407(730)	71(147)	265(481)	416(703)	61(135)	271(473)
mHSC-L	2939(3463)	438(519)	1021(1198)	151(171)	25(30)	103(116)	76(87)	5(6)	56(61)
mESC	19803(28634)	2956(4273)	6854(9888)	3733(4355)	707(861)	2453(2834)	4202(4587)	822(917)	2738(2975)
hESC	3031(4726)	454(714)	1060(1644)	1855(2481)	362(510)	1224(1626)	2325(2791)	415(540)	1517(1818)
hHEP	6641(10410)	998(1555)	2300(3593)	2222(2881)	439(595)	1468(1881)	4053(4849)	827(1021)	2643(3133)
mDC	498(790)	74(119)	184(284)	1664(2114)	316(427)	1087(1377)	2600(3182)	526(665)	1689(2051)

Table S2. Top-10 gene pairs predicted by GENELink on hESC dataset.

TF	Target gene	Reference
TFAP2A	KLF6	Harmonizome (Rouillard et al., 2016)
TFAP2A	MEIS1	Harmonizome (Rouillard et al., 2016)
TFAP2A	EPHA1	ChIPBase (Huang et al., 2023)
TFAP2A	GAD1	
TFAP2A	PDCD11	
TFAP2A	PSMC3IP	ChIPBase (Huang et al., 2023)
TFAP2A	SHISA6	ChIPBase (Huang et al., 2023)
TFAP2A	FAM46A	ChIPBase (Huang et al., 2023)
TFAP2A	TMEM48	ChIPBase (Huang et al., 2023)
TFAP2A	MLF1IP	ChIPBase (Huang et al., 2023)

References

G. Chen and Z.-P. Liu. Graph attention network for link prediction of gene regulations from single-cell rna-sequencing data. *Bioinformatics*, 38(19):4522–4529, 2022.

J. Huang, W. Zheng, P. Zhang, Q. Lin, Z. Chen, J. Xuan, C. Liu, D. Wu, Q. Huang, L. Zheng, et al. Chipbase v3. 0: the encyclopedia of transcriptional regulations of non-coding rnas and protein-coding genes. *Nucleic Acids Research*, 51(D1):D46–D56, 2023.

V. A. Huynh-Thu, A. Irrthum, L. Wehenkel, and P. Geurts. Inferring regulatory networks from expression data using tree-based methods. *PloS one*, 5(9):e12776, 2010.

K. Kc, R. Li, F. Cui, Q. Yu, and A. R. Haake. Gne: a deep learning framework for gene network inference by aggregating biological information. *BMC systems biology*, 13:1–14, 2019.

T. Moerman, S. Aibar Santos, C. Bravo González-Blas, J. Simm, Y. Moreau, J. Aerts, and S. Aerts. Grnboost2 and arboreto: efficient and scalable inference of gene regulatory networks. *Bioinformatics*, 35(12):2159–2161, 2019.

A. Pratapa, A. P. Jalihal, J. N. Law, A. Bharadwaj, and T. Murali. Benchmarking algorithms for gene regulatory network inference from single-cell transcriptomic data. *Nature methods*, 17(2):147–154, 2020.

A. D. Rouillard, G. W. Gundersen, N. F. Fernandez, Z. Wang, C. D. Monteiro, M. G. McDermott, and A. Ma'ayan. The harmonizome: a collection of processed datasets gathered to serve and mine knowledge about genes and proteins. *Database*, 2016, 2016.

H. Shu, J. Zhou, Q. Lian, H. Li, D. Zhao, J. Zeng, and J. Ma. Modeling gene regulatory networks using neural network architectures. *Nature Computational Science*, 1(7):491–501, 2021.

P. Velickovic, G. Cucurull, A. Casanova, A. Romero, P. Lio, Y. Bengio, et al. Graph attention networks. *stat*, 1050(20):10–48550, 2017.

Z. Yang, M. Ding, X. Zou, J. Tang, B. Xu, C. Zhou, and H. Yang. Region or global? a principle for negative sampling in graph-based recommendation. *IEEE Transactions on Knowledge and Data Engineering*, 35(6):6264–6277, 2022.

Y. Yuan and Z. Bar-Joseph. Deep learning for inferring gene relationships from single-cell expression data. *Proceedings of the National Academy of Sciences*, 116(52):27151–27158, 2019.
